# Loss of *Foxm1* Results in Reduced Somatotrope Cell Number during Mouse Embryogenesis

**DOI:** 10.1371/journal.pone.0128942

**Published:** 2015-06-15

**Authors:** Michael J. Calderon, Adam G. Ploegman, Brock Bailey, Deborah O. Jung, Amy M. Navratil, Buffy S. Ellsworth

**Affiliations:** 1 Department of Physiology, Southern Illinois University, Carbondale, Illinois, United States of America; 2 Department of Zoology and Physiology, University of Wyoming, Laramie, Wyoming, United States of America; Queen Mary University of London, UNITED KINGDOM

## Abstract

FOXM1, a member of the forkhead box transcription factor family, plays a key role in cell cycling progression by regulating the expression of critical G1/S and G2/M phase transition genes. In vivo studies reveal that *Foxm1* null mice have a 91% lethality rate at e18.5 due to significant cardiovascular and hepatic hypoplasia. Thus, FOXM1 has emerged as a key protein regulating mitotic division and cell proliferation necessary for embryogenesis. In the current study, we assess the requirement for *Foxm1* in the developing pituitary gland. We find that *Foxm1* is expressed in the pituitary at embryonic days 10.5-e18.5 and localizes with markers for active cell proliferation (BrdU). Interestingly, direct analysis of *Foxm1* null mice at various embryonic ages, reveals no difference in gross pituitary morphology or cell proliferation. We do observe a downward trend in overall pituitary cell number and a small reduction in pituitary size in e18.5 embryos suggesting there may be subtle changes in pituitary proliferation not detected with our proliferation makers. Consistent with this, *Foxm1* null mice have reductions in both the somatotrope and gonadotrope cell populations.

## Introduction

The development of the anterior pituitary gland involves a highly orchestrated cascade of morphogenetic signals and transcription factors that give rise to five hormone-secreting cell lineages in a characteristic spatial and temporal manner. These endocrine cell lineages are of primary importance in the regulation of critical physiological functions including control of metabolism, growth, lactation, and reproduction. Despite recent progress defining the transcriptional mechanisms that underlie pituitary organogenesis and cell differentiation, our understanding of these mechanisms is far from complete. [1, 2].

Forkhead box (FOX) transcription factors are a family of proteins that share a highly conserved DNA binding domain [3] and control a range of functions in the cell, including development [4, 5], cell cycle control [6, 7], cell survival [8–10], metabolism [11, 12], and immunoregulation [13, 14]. Interestingly, recent work has highlighted the observation that several forkhead factors affect both pituitary cell differentiation and function. The impact of forkhead proteins on pituitary development was made strikingly clear with the initial discovery that FOXL2 [15] regulates several genes necessary for gonadotrope function, including those that code for gonadotropin-releasing hormone receptor, *Cga*, and *Fshb* [16–20]. Indeed, without FOXL2, FSH production is severely impaired, causing infertility [21]. FOXP3, typically thought to be involved in the development and regulation of T cells, is not expressed the pituitary gland, but is important for maintaining appropriate gonadotropin levels [22]. A mutation in *Foxp3* results in severely reduced *Lhb* and *Fshb* levels, leading to infertility [22–24]. FOXD1, while also not expressed in the pituitary, appears essential for up regulation of genes necessary for LH production [25]. In contrast, FOXA1 is part of a complex that represses *Gh1* expression [26]. Similarly, FOXO1 represses *Lhb* expression in studies using immortalized cell lines [27]. Thus, there is clear evidence that forkhead factors play a critical role in pituitary function. However, the role of the proliferation-associated forkhead transcription factor, FOXM1, within the pituitary remains largely undefined.

FOXM1 (Accession No. NM_008021) is a transcription factor that plays an essential role in organogenesis by controlling expression of genes involved in cell cycle progression and mitotic division. Given the critical role of cell proliferation in organ development, it is not surprising that deletion of *Foxm1* results in 91% lethality by embryonic day (e)18.5 [28]. The high lethality can be attributed to significant cardiovascular anomalies including myocardium defects and ventricular hypoplasia [29]. In addition, hepatoblasts of the liver fail to enter mitosis [28], and severe abnormalities of pulmonary vasculature are also detected in *Foxm1* null animals[30].

Previous work has found that FOXM1 is expressed in the embryonic pituitary [31]. Thus, we were intrigued with the possibility that FOXM1 plays an important role in cell proliferation necessary for pituitary development. Here we compare pituitary morphology, hormone production and cell proliferation of mouse embryos deficient in *Foxm1* with their wild type littermates. We observe no difference in proliferation, but a reduction the number of somatotropes and gonadotropes is apparent.

## Materials and Methods

### Mice

*Foxm1* knockout mice were graciously provided by Dr. Raychaudhuri, University of Illinois at Chicago, and maintained on a C57BL/6J background [28]. Mice were kept under a 12-hour dark-light cycle and fed Purina Mills Formulab diet 5008 ad libitum. The morning the copulatory plug was detected was considered to be embryonic day 0.5 (e0.5). Embryos were obtained from mating of *Foxm1* ^*+/-*^ mice. Littermates were used for all experiments in which normal and mutant embryos were compared. Genotyping for the wild type *Foxm1* allele was performed using quantitative polymerase chain reaction (qPCR) with the following primers: 5’-ATGTTGACACCAGGCCTACCAGAA and 5’-TATGTGTGGAACGCAGGAAGGTGA. To genotype the null allele, polymerase chain reaction (PCR) was used with the following primers: 5’-TGGCTTCCCAGCAGTACAAATC and 5’- TCTCGCTCAATTCCAAGACCAG.

The Southern Illinois University Animal Care and Use Committee approved all procedures using mice (Animal Assurance Number A-3078-01). All experiments were conducted in accord with the principles and procedures outlined in the NIH Guidelines for the Care and Use of Experimental Animals.

### Histology and Immunohistochemistry

To detect cell proliferation in embryonic pituitaries, pregnant mice were given an intraperitoneal injection of bromodeoxyuridine (BrdU) at 100 μg/g body weight 2 hours before the embryos were collected [32]. Pregnant dams were euthanized by CO_2_ inhalation and embryos were dissected from the uterus. Embryos were euthanized by submerging in ice cold PBS.

Embryos were fixed in 4% formaldehyde in phosphate buffered saline (PBS; pH 7.2) for 45 minutes to 24 hours (depending on the stage of development). Any embryos that were discolored or underdeveloped as compared to littermates were considered dead and were not included in our analyses. All samples were then washed in PBS, dehydrated in a graded series of ethanol and embedded in paraffin. Five micron sections were deparaffinized in xylene, rehydrated through a series of graded ethanol washes. Mid-coronal sections that were comparable between wild type and mutants were chosen for immunohistochemistry to label the various hormone-secreting cell types at e17.5 and e18.5. Due to the small size of early stage pituitaries, mid-sagittal, rather than coronal, sections were chosen for pituitary stages prior to e17.5.

To visualize BrdU, FOXM1 and phosphohistone H3 (pHH3), endogenous peroxidases were removed with 1.5% hydrogen peroxide in water. Epitopes were then unmasked by boiling in 10mM citric acid for 10 minutes followed by a 20 minute cool down. The specimens were blocked using the blocking solution from the tyramide signal amplification (TSA) fluorescein isothiocyanate (FITC) kit (Perkin-Elmer Life Sciences, Boston, MA) for 10 minutes then incubated with primary antibodies for BrdU (Invitrogen, Boston, MA, mouse, 1:100), FOXM1 (Santa Cruz Biotechnology, Santa Cruz, CA, rabbit, 1:30) or pHH3 (Millipore, Temecula, CA, rabbit, 1:100) overnight at 4°C (Table 1). BrdU specimens were then incubated with the appropriate anti-mouse biotinylated secondary antibodies (Vector, Burlingame, CA, goat, 1:200) for 30 minutes while FOXM1 and pHH3 specimens were incubated with bioinylated anti-rabbit antibodies (Jackson, West Grove, PA, goat, 1:200). All specimens were incubated in streptavidin-horseradish peroxidase (Perkin-Elmer) for 30 minutes, followed by a 10 minute incubation in FITC (Perkin-Elmer). The sections were finally incubated for 5 minutes with 4’,6-diamidino-2-phenylindole (DAPI) (1:100; 167 nM, Molecular Probes, www.invitrogen.com) and mounted using Aquamount (Lerner Laboratories, Pittsburgh, PA).

**Table 1:** Antibody Information.

Name	Clonality	Host	Supplier	Cat. No.	Immunogen	Registry ID
FOXM1	polyclonal	rabbit	Santa Cruz	sc-502	C-terminus of human FOXM1	AB_631523
BrdU	monoclonal	mouse	Life Technologies	B35128	5-iodouridine	AB_10562901
p57	polyclonal	rabbit	Santa Cruz	sc-8298	aa 45–135 of human p57	AB_2078155
pHH3	polyclonal	rabbit	Millipore	06–570	human pHH3 (Ser10)	AB_310177
pERK	monoclonal	rabbit	Cell Signaling	4370	Thr202/Tyr204 of human p44 MAP kinase	AB_2315112
ACTH	polyclonal	rabbit	NHPP	N/A	rat ACTH	AB_2313902
TSHB	polyclonal	rabbit	NHPP	N/A	rat TSHB	N/A
GH	polyclonal	rabbit	NHPP	N/A	mouse GH	N/A
CGA	polyclonal	rabbit	NHPP	N/A	rat CGA	N/A
LHB	polyclonal	guinea pig	NHPP	N/A	rat LHB	N/A
biotin anti-mouse	polyclonal	goat	Vector Laboratories	BA-9200	mouse	AB_2336171
biotin anti-rabbit	polyclonal	goat	Jackson Laboratories	111-067-003	rabbit	AB_2337971
alexa fluor 488 anti-rabbit	polyclonal	goat	Molecular Probes	A-11008	rabbit	AB_143165
TRITC-anti-guinea pig	polyclonal	donkey	Jackson Laboratories	706-026-148	guinea pig	AB_2340446
TRITC-anti-rabbit	polyclonal	goat	Jackson Laboratories	111-297-003	rabbit	AB_2338034

Double immunohistochemistry for FOXM1 with BrdU or pHH3, was performed by detecting FOXM1 as described above. Tissue sections were then blocked for 15 minute with streptavidin and biotin block (Vector Laboratories, 1:50) as well as with anti-rabbit Fab fragment according to manufacturer’s instructions and BrdU or pHH3 was detected as described above, except that sections were incubated with TRITC (Perkin-Elmer) rather than FITC. Controls in which each primary antibody was omitted were performed and revealed minimal cross-reactivity between stains (data not shown).

For immunofluorescent labeling of p-ERK1/2, pituitary sections were fixed, embedded, and epitopes unmasked as described above. Sections were washed in TBS and blocked with 10% normal goat serum/2x casein in TBS for 30 min at room temperature. Sections were then incubated in anti-p-ERK1/2 primary antibody diluted 1:80 in TBS/1× casein overnight; additionally normal rabbit IgG at equivalent concentration (micrograms/ml) was used as negative control. After overnight primary antibody incubation, sections were washed with TBS and incubated with Alexa Fluor 488 anti-rabbit secondary antibody (1:100) for 20 min at room temperature in the dark. Slides were then washed and mounted in Vectashield with DAPI. Images were obtained on a Zeiss 710 Laser Scanning Confocal Microscope using the appropriate filters.

To visualize adrenocorticotropic hormone (ACTH), thyroid stimulating hormone (TSHB), growth hormone (GH), the common α-subunit (CGA) and luteinizing hormone (LHB), tissue sections were blocked with TSA blocking solution (Perkin-Elmer Life Sciences) and incubated with the appropriate antibody (National Hormone and Peptide Program, Torrance, CA) for one hour at room temperature. Tissue sections were incubated with anti-guinea pig or anti-rabbit secondary antibodies that are directly conjugated to TRITC (Jackson Immunoresearch West Grove, PA, 1:100) for 30 minutes to detect LHB or ACTH, GH, TSHB and CGA, respectively. Sections were stained with DAPI and mounted as above.

Pituitary morphology was observed by staining tissue sections with hematoxylin and eosin. Briefly, paraffin was melted and then removed with two washes of citraclear. The sections were then washed in three changes of 100% ethanol for two minutes each, wash in distilled water for two minutes, incubated in filtered hematoxylin for 4 minutes then washed in distilled water. Sections were dipped in acid alcohol and then lithium carbonate followed by a rinse in distilled water and then dipped in eosin 1–5 times. Sections were placed in 100% isopropyl alcohol three times for 2 minutes each before mounting.

Digital images were captured with a Leica DM 5000B fluorescent microscope and Retiga 2000R digital camera. DAPI and the corresponding FITC and/or TRITC pictures were merged using Adobe Photoshop CS3.

### Histological Quantification

Phosphohistone H3-positive cells were separated into dimly fluorescent cells, which mark cells in late G2 phase, and brightly stained cells, which mark cells in M phase [33]. The total number of pituitary cells was counted by labeling cell nuclei with DAPI. A ratio of cells in late G2 or in M phase to total DAPI count was calculated for each pituitary. Four to five littermate pairs were analyzed and one section was counted per pituitary.

The total number of BrdU-positive and DAPI-positive cells per tissue section were counted at ages e10.5, e12.5 and e14.5. The entire pituitary could be viewed at 200x magnification at all ages. A ratio of positive cells to total DAPI count was calculated for each pituitary. Four to five littermate pairs were analyzed and one section was counted per pituitary.

LHB, GH, TSHB, CGA and ACTH-positive cells and DAPI-positive cells were counted manually at e17.5 and e18.5. The entire pituitary could not be viewed at 200x magnification, so only half of the pituitary was counted. Only the intermediate and anterior lobes were counted. A ratio of positive cells to total DAPI count was calculated for each pituitary. The ratios were then standardized to the wild type littermate. Four to seven littermate pairs were analyzed and one section was counted per pituitary.

Area was determined for approximately three sections per pituitary using ImageJ64. The anterior pituitary was outlined in ImageJ64 and area was measured in μm^2^. Values were set relative to wild type controls for each littermate pair. Four littermate pairs were analyzed for the e12.5 age group and eight littermate pairs were analyzed for the e14.5 and e18.5 age groups.

### Quantitative RT-PCR

Pituitary glands were dissected from e17.5 and e18.5 embryos. RNA was then isolated using the RNAqueous-Mcro kit (Ambion, Inc.) according to manufacturer’s instructions. RNA was treated with DNase I and DNase inactivating reagent from the TURBO DNase-free kit (Ambion, Inc.) as per manufacturer’s instructions. RNA concentrations were determined by spectrophotometry at 260nm. Ten ng of total RNA was used with the TaqMan RNA-to-CT 1-step Kit and TaqMan probes. Amplification was accomplished using Taqman Gene Expression Assays (Applied Biosystems) as per manufacturer’s instructions: *Cga* (Accession No. NM_009889)(Mm01209400_m1), *Gh1* (Accession No. NM_008117)(Mm00433590_g1), *Pomc* (NM_001278581)(Mm00435874_m1), *Lhb* (Accession No. NM_008497)(Mm00656868_g1) and *Tshb* (Accession No. (Accession No. BC144732)(Mm00437190_m1), utilizing *Actb* (Accession No. NM_007393)(4352933E) as the endogenous control (all Taqman probes from Applied Biosystems, Inc.). All experiments were performed in triplicate. Real time RT-PCR was performed on a CFX96 Real Time System (BioRad). Experiments were run at 48°C for 15 minutes to synthesize the cDNA followed by 10 minutes at 95°C to deactivate the reverse transcriptase and activate the *Taq* polymerase and then 40 cycles of 95°C for 15 seconds and 60°C for 1 minute for amplification. No-template controls and no-reverse transcriptase controls were used to assure the absence of contamination and efficacy of the DNase treatment, respectively. Melt curve analysis was performed to ensure that no primer-dimer amplification occurred. Data were analyzed by the ΔΔC_T_ method [34, 35]. The values for ΔΔC_T_ were calculated by subtracting the average ΔC_T_ of wild type controls from the ΔC_T_ for each sample. C_T_ values over 30 were considered unreliable and were not included in our analyses.

### Statistical Analysis

All results are expressed as mean ± SEM. Data were analyzed by Student’s *t*-test using Microsoft Excel. P-values less than 0.05 were considered significant (*).

## Results

### FOXM1 Expression During Pituitary Development

**Fig 1:**
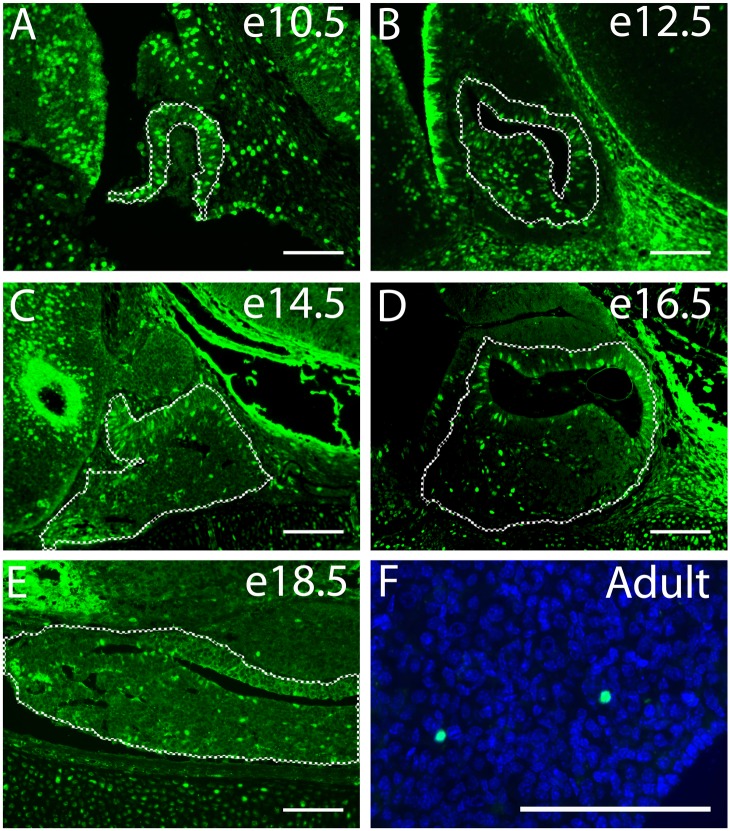
The number of FOXM1-positive cells decreases as pituitary development progresses. Immunohistochemistry for FOXM1 was performed to identify its expression pattern in murine pituitary gland at (A) embryonic day 10.5 (e10.5), (B) e12.5, (C) e14.5, (D) e16.5 and (E) e18.5 as well as in (F) adult pituitary. All specimens are midsagittal sections (A-D), except for e18.5 and adult sections (E, F), which are oriented coronally. FOXM1 is most abundant in the early stages of development (A, B), when cell proliferation is greatest. As proliferation slows and differentiation begins at e16.5 and e18.5 (D,E), the number of FOXM1 positive cells is reduced. The number is even further reduced in the mature pituitary (F), as the differentiated cells have left the cell cycle. Scale bar represents 100 μm.

**Fig 2:**
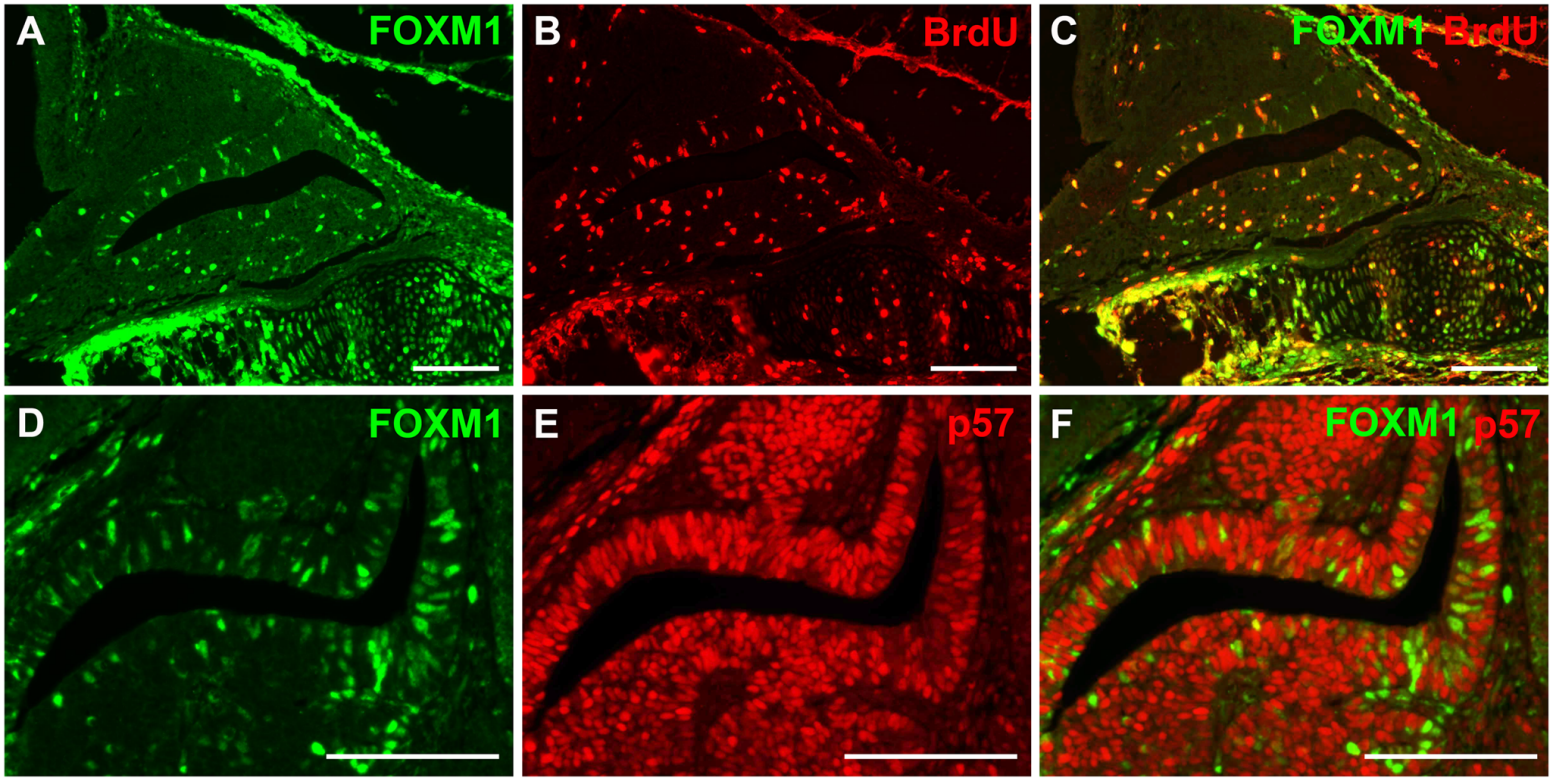
FOXM1 is present in actively proliferating cells. FOXM1 (green) and a marker for cells in S phase, BrdU (red), were labeled in midsagittal pituitary sections from wild type e16.5 mouse embryos (A-C). FOXM1 (green) and a marker for non-cycling precursors, p57 (red), were labeled in midsagittal pituitary sections from wild type e14.5 mouse embryos (D-F). Scale bar represents 100 μm.

To begin identifying the role that FOXM1 plays in anterior pituitary organogenesis, we first examined the spatial and temporal distribution of FOXM1 in the normal mouse pituitary over the course of embryonic development and in adulthood using immunohistochemistry. While FOXM1 was present at all ages examined, the proportion of FOXM1 immuno-positive nuclei dropped as development progressed. At early embryonic ages (Fig 1A and 1B), a large percentage of the cells in the nascent pituitary contain the FOXM1 protein, consistent with high levels of cell proliferation at these stages of development. With the beginning of terminal differentiation in the pituitary, a reduction in FOXM1 positive cells was seen at e14.5 (Fig 1C–1F) [1, 36, 37]. To directly test whether FOXM1 localizes to proliferating cells, we performed double immunohistochemistry on wild type mice at e16.5, for FOXM1 and BrdU (Fig 2A–2C). We found that within the pituitary, FOXM1 consistently co-localizes with BrdU. Non-cycling precursors were marked by labeling p57 [38]. We found that FOXM1 is not present in cells that have left the cell cycle, consistent with its role as a promoter of cell cycle progression (Fig 2D–2F).

### Pituitary Morphology and Cell Proliferation in *Foxm1*-Deficient Embryos

**Fig 3:**
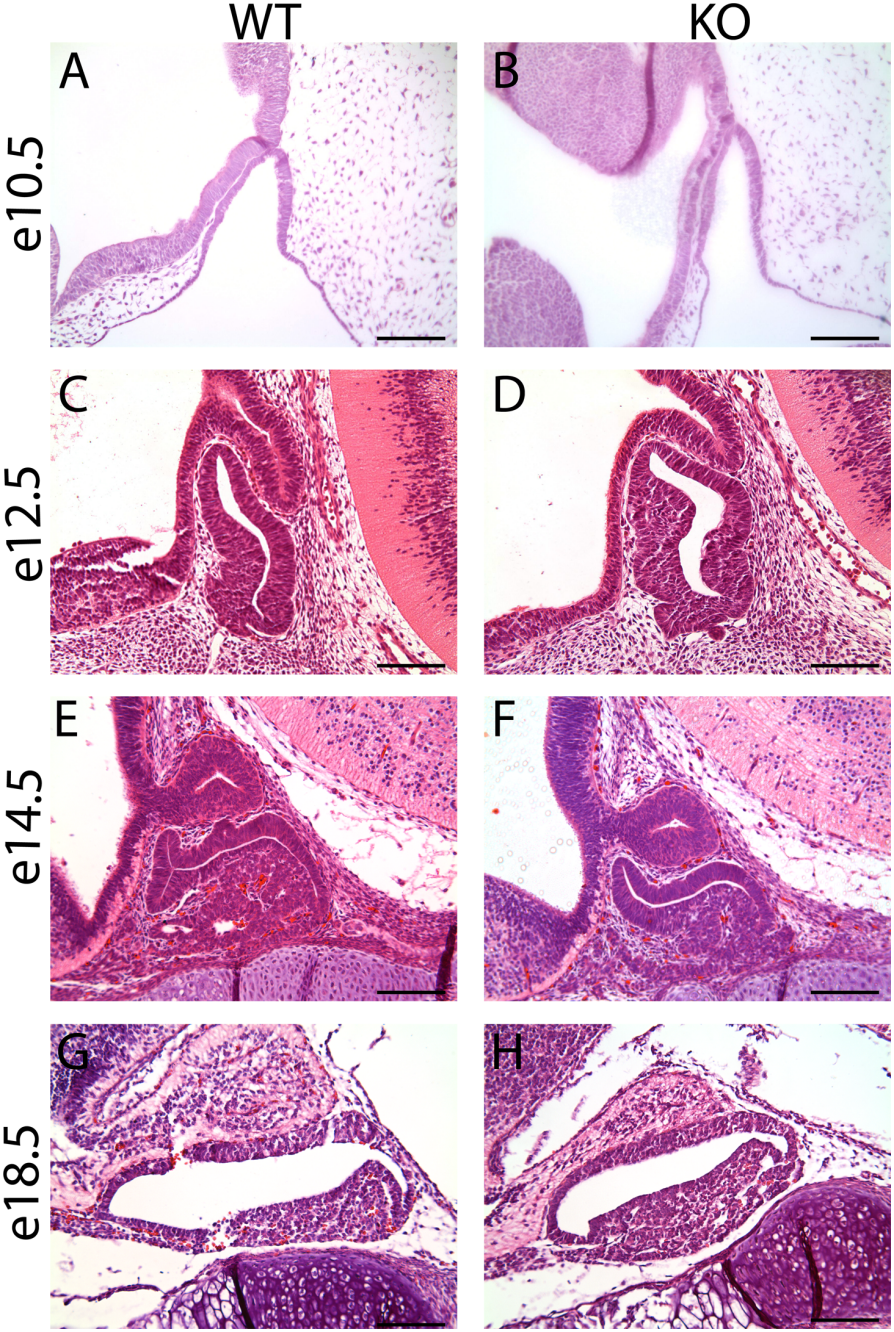
*Foxm1* ^*-/-*^ pituitary glands exhibit normal morphology. Pituitary glands of *Foxm1* ^*-/-*^ embryos (KO) and wild type littermates (WT) were stained with hematoxylin and eosin at (A-B) embryonic day 10.5 (e10.5), (C-D) e12.5, (E-F) e14.5 and (G-H) e18.5. No gross morphological differences were seen between the pairs. Scale bar represents 100 μm.

Our data support the conclusion that FOXM1 is present in actively dividing pituitary cells during early stages of development, thus we hypothesized that deletion of *Foxm1* would lead to significant malformation of Rathke’s pouch. If correct, we would expect changes in gross pituitary morphology. To test this notion, we compared wild type embryos stained with hematoxylin and eosin with their *Foxm1* null littermates (Fig 3). Surprisingly, morphological analysis did not reveal any overt changes in overall pituitary structure.

**Fig 4:**
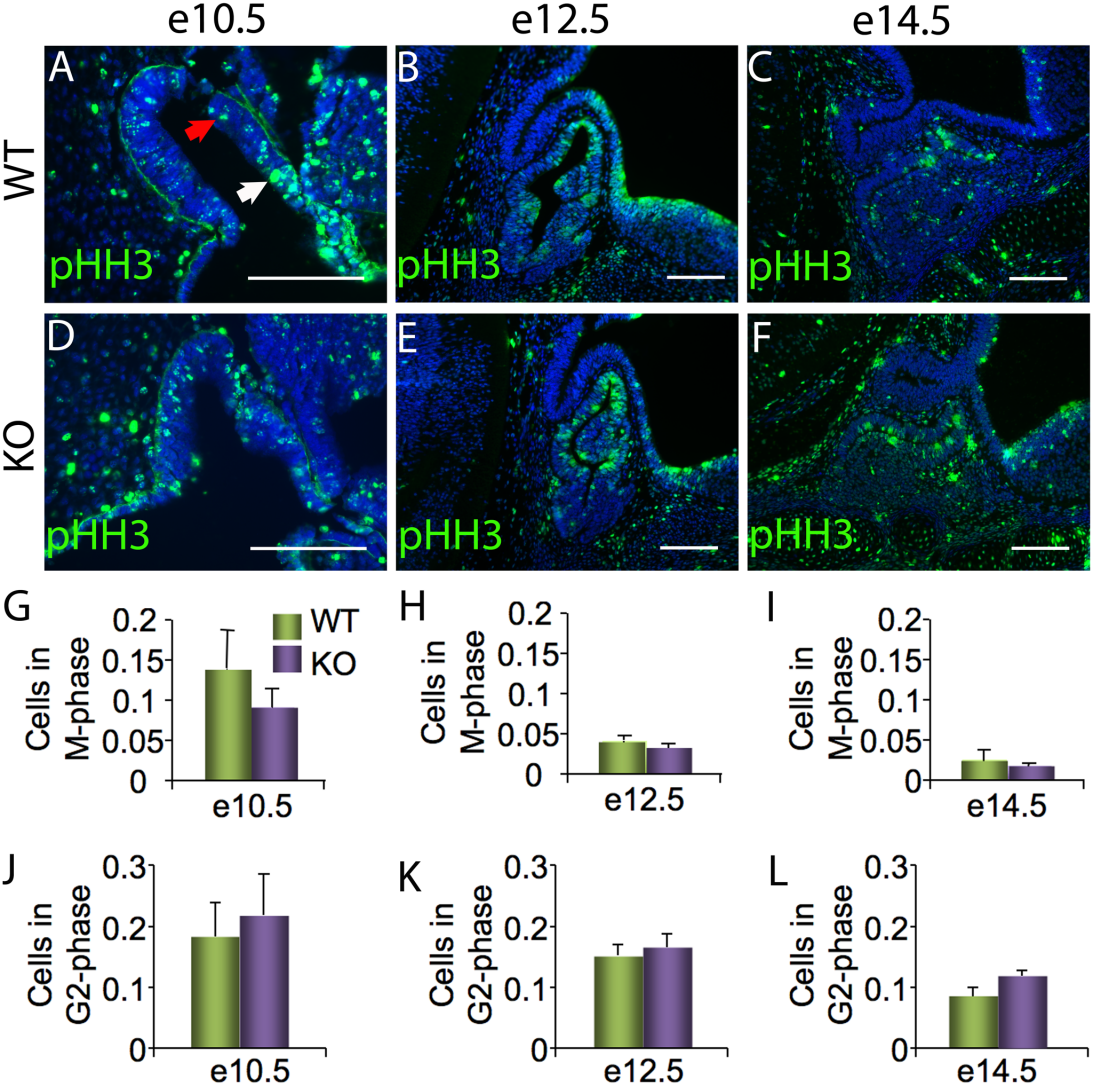
No significant difference in M phase or G2 phase is apparent in *Foxm1* ^*-/-*^ pituitary glands. Immunohistochemistry was performed on midsagittal pituitaries from *Foxm1* ^*-/-*^ embryos (KO) and wild type littermates (WT) at embryonic day 10.5 (e10.5), e12.5, and e14.5 embryos to identify cellular proliferation. (A-F) Staining of phosphohistone H3 (pHH3, green) allows for differentiation between cells in M phase (brightly stained, white arrow) and G2 phase (dimly stained; red arrow). Cells in M phase, G2 phase and total cells per pituitary section were counted for each age and genotype. Graphs represent the ratio of cells in (G-I) M phase or (J-L) G2 phase to total DAPI (blue) counts per pituitary section. Data are expressed as mean ± SEM of four or five littermate pairs for each age. The data were analyzed by Student *t*-test to determine significant difference between WT and KO.

Despite no change in morphology, at issue is the level of cell proliferation within pituitary from *Foxm1* null embryos. To directly test this, proliferation of *Foxm1* null embryos and their wild type littermates was compared using pHH3. Not only is pHH3 a proliferation marker, it can also be used to differentiate between cells in late G2 phase and M phase. G2 cells have significantly fewer histones phosphorylated and so stain more dimly than cell in M phase, which are brightly stained due to the increased phosphorylated H3 subunits [33]. When *Foxm1* null embryos were compared to their wild type littermates, there was no statistical difference in the number of G2 phase cells, M phase cells or total pHH3 positive cells at e10.5, e12.5 or e14.5 (Fig 4A–4L). Extracelluar signal regulated kinase (ERK), promotes proliferation in many tissues and its activity is sustained by FOXM1 in hepatocellular carcinoma cells [39, 40]. Consistent with no changes in pituitary cell proliferation, we also observed no difference in activity of ERK in *Foxm1* null embryos at e10.5 (data not shown).

**Fig 5:**
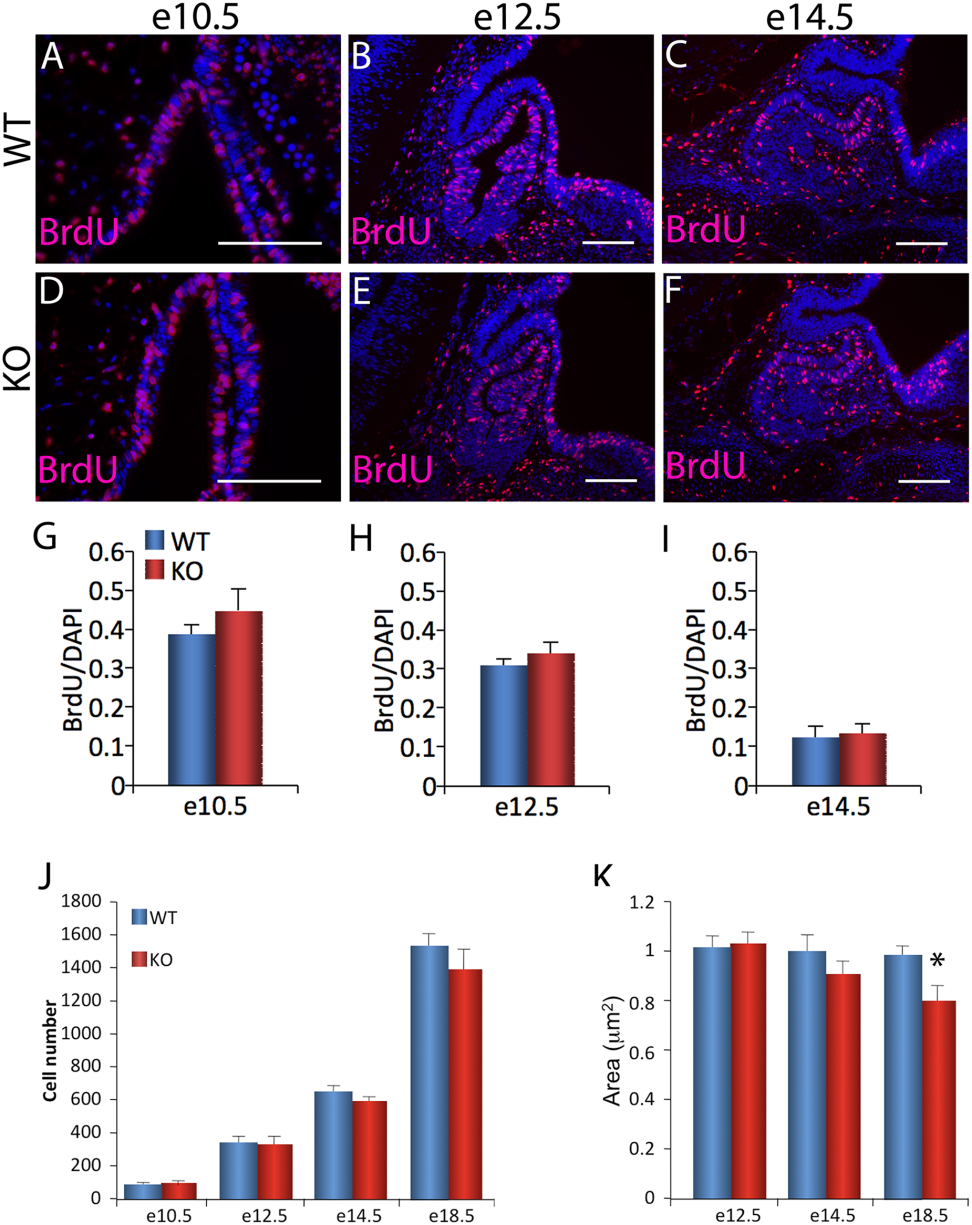
No significant difference in S phase is apparent in *Foxm1* ^*-/-*^ pituitary glands. (A-F) BrdU (red) was used as a marker for cells in S phase and DAPI (blue) labels all cell nuclei. Scale bar represents 100 μm. (G-I) A ratio of BrdU-positive cells to total DAPI count per pituitary section was calculated for *Foxm1* ^*-/-*^ embryos (KO) and wild type littermates (WT) at each age. Data are expressed as mean ± SEM of four or five littermate pairs for each age. The data were analyzed by Student *t*-test to determine significant difference between WT and KO. (J) The total number of cells per pituitary section was counted for embryonic day 10.5 (e10.5), e12.5, e14.5, e18.5 in *Foxm1* ^*-/-*^ (KO) and wild type littermates (WT). Data are expressed as mean ± SEM of six littermate pairs for each age. The data were analyzed by Student *t*-test to determine significant difference between WT and KO. (K) Area of approximately three pituitary sections per individual was measured. Values are shown relative to WT littermate controls. Data are expressed as mean ± SEM of four littermate pairs for e12.5 and eight littermate pairs for e14.5 and e18.5. The data were analyzed by Student *t*-test to determine significant difference between WT and KO (*P<0.05).

As an alternative proliferation marker, BrdU was also analyzed. Again, no significant difference in the number of BrdU-positive cells in the pituitary gland was observed between *Foxm1* null embryos and wild type littermates at e10.5, e12.5 or e14.5 (Fig 5A–5I). In agreement with the literature, the percent of cells staining for BrdU in the pituitary fell with increasing age (data not shown). Additionally, pituitary sections from wild type and *Foxm1* null embryos were labeled with the proliferation marker, Ki67. No significant difference in the number of Ki67-immunopositive cells was observed between genotypes (data not shown).

Our data suggests there are no changes in anterior pituitary cell proliferation and morphology in *Foxm1* null embryos, therefore we next sought to confirm our results by quantitatively analyzing the pituitary cell density. Pituitary sections were labeled with DAPI and stained cells were quantitated. We observed no significant difference between the number of cells per pituitary section between *Foxm1* null embryos and their wild type littermates at e10.5, e12.5, e14.5 and e18.5, although there was a trend toward decreased cell number in *Foxm1* null pituitary glands at later ages (Fig 5J).

To more carefully analyze pituitary size, the area of pituitary tissue sections was measured at e12.5, e14.5 and e18.5 (Fig 5K). *Foxm1* null embryos were not significantly smaller at e12.5 or e14.5, although there was a downward trend in area of pituitary sections at e14.5. At e18.5 there was a small but significantly decrease in area of pituitary sections in *Foxm1* null embryos as compared to wild type littermate controls, suggesting that *Foxm1* is required for normal growth of the embryonic pituitary gland.

### Hormone Production in *Foxm1*-Deficient Embryos

**Fig 6:**
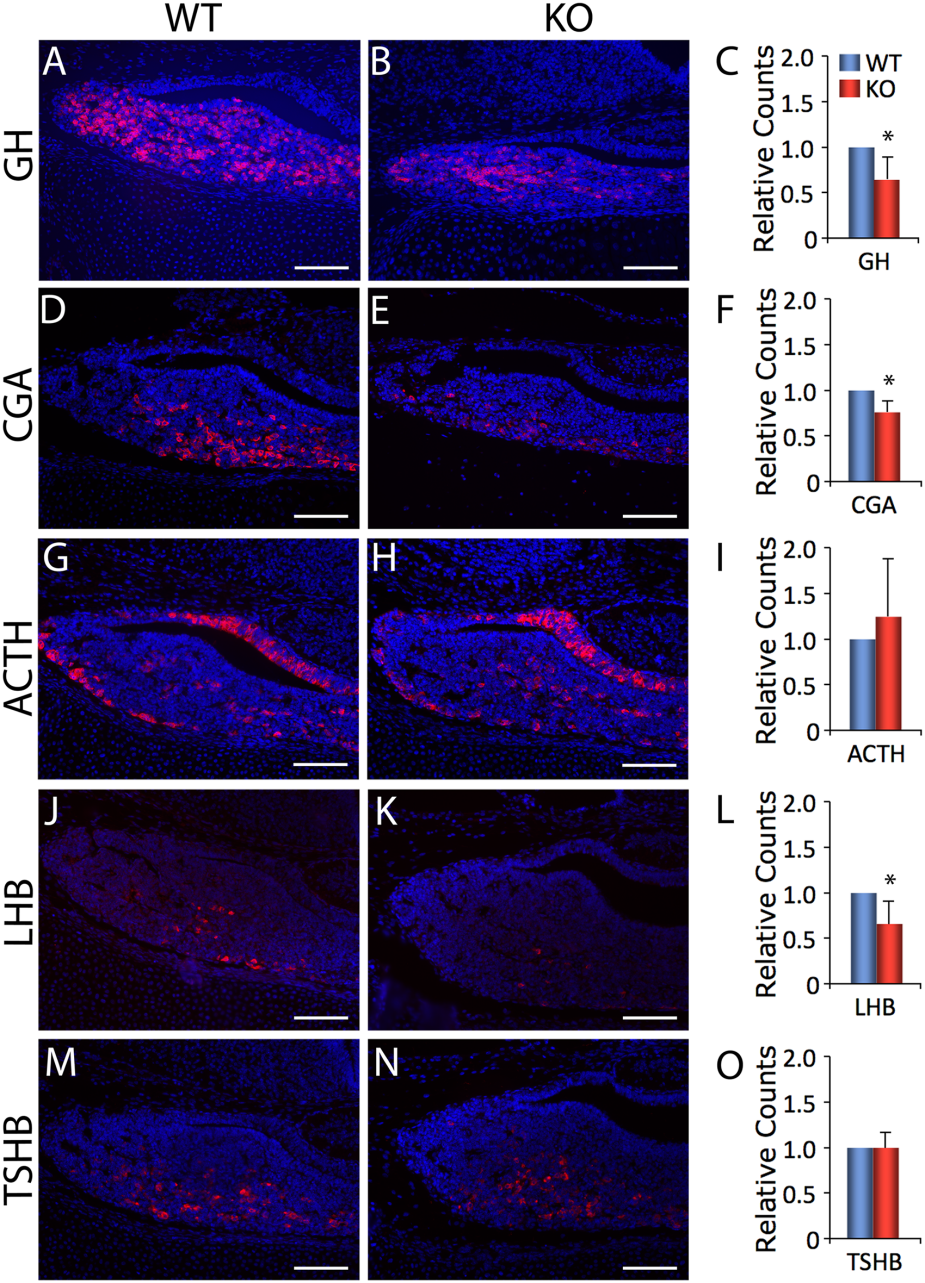
The number of cells containing GH, LHB, and CGA are reduced in *Foxm1* ^*-/-*^ pituitary glands. Immunohistochemistry for (A-B) GH (red), (D-E) CGA (red), (G-H) ACTH (red), (J-K) LHB (red), (M-N) TSHB (red) was performed on e17.5/18.5 coronal sections. DAPI (blue) labels all cell nuclei. Scale bar represents 100 μm. The total number of cells containing (C) GH, (F) CGA, (I) ACTH, (L) LHB, (O) TSHB and total DAPI in each pituitary were counted for *Foxm1* ^*-/-*^ embryos (KO) and wild type (WT) littermates. The KO ratio (hormone/DAPI) was then normalized to the WT ratio. Data are expressed as mean ± SEM of 4–6 littermate pairs for each age. The data were analyzed by Student *t*-test to determine significant difference between WT and KO (*P < 0.05).

To determine if *Foxm1* is required for normal pituitary cell lineage commitments, immunohistochemistry was performed on e17.5-e18.5 embryos deficient in *Foxm1* or their wild type littermates for markers of the 5 endocrine cell types. Individual cell types from *Foxm1* null embryos and wild type littermates were counted and expressed as a ratio of the total number of pituitary cells (Fig 6A–6O). Interestingly, the number of GH-, CGA- and LHB-positive cells was significantly reduced in *Foxm1* null embryos, suggesting that *Foxm1* is required for normal numbers of somatotropes and gonadotropes to differentiate. No significant difference was observed in the number of ACTH- or TSHB-positive cells.

**Fig 7:**
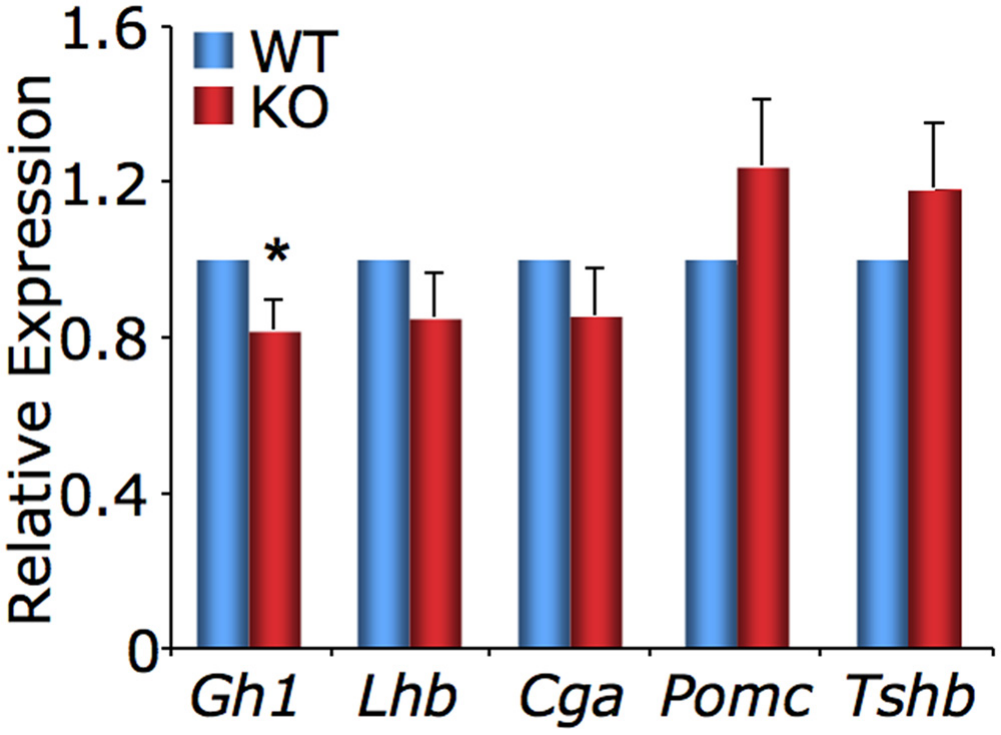
Expression of *Gh1* is reduced in *Foxm1* ^*-/-*^ pituitary glands. Real time RT-PCR for the genes encoding growth hormone (*Gh1*), luteinizing hormone **β** (*Lhb*), the common α subunit (*Cga*), the precursor for ACTH (*Pomc*) and thyroid stimulating hormone (*Tshb*) was performed on RNA isolated from pituitary of e17.5 and e18.5 *Foxm1* ^*-/-*^ embryos (KO) and wild type littermates (WT). Values were calculated using the ΔΔC_T_ method and normalized to WT. Data are expressed as mean ± SEM of 7 littermate pairs for each age and were analyzed by Student *t*-test to determine significant difference between WT and KO (*P < 0.05).

To more accurately quantify hormone expression in *Foxm1* null embryos, we employed quantitative reverse transcription polymerase chain reaction (qRT-PCR). Consistent with a reduced number of somatotropes, expression of the gene encoding for GH (*Gh1*) was modestly but significantly reduced in *Foxm1* null embryos as compared to wild type littermates (Fig 7). While expression of *Lhb* and *Cga* showed a trend toward being reduced in *Foxm1* null embryos, this was not statistically significant. While not statistically significant, an upward trend was observed in expression of *Pomc* or *Tshb*. We did not assess prolactin or follicle-stimulating hormone mRNA levels because they are present in such low quantities in the embryo it is very difficult to measure reliably [41].

## Discussion

Previous work has highlighted the essential role of FOXM1 in cell cycle progression in various tissues [28, 42] although its role in pituitary organogenesis remains unknown. We find that FOXM1 is present in actively proliferating pituitary cells during development consistent with a role in promoting proliferation during pituitary development (Fig 1). Interestingly, when we assessed the number of proliferating cells in in the pituitary glands of *Foxm1* null embryos, we saw no statistical difference in proliferation at any age analyzed (Figs 4 and 5). However, we did observe a downward trend in the number of cells at e18.5 (Fig 5J). Additionally, the area of *Foxm1* ^*-/-*^ pituitaries is significantly reduced at e18.5 (Fig 5K). These data, together with the fact that we observe a decrease in the number of somatotropes and gonadotropes, (Figs 6 and 7) suggest there may be a subtle defect in pituitary cell proliferation in *Foxm1* null embryos that is not statistically significant. If correct, it would suggest a differential requirement of FOXM1 for proliferation among precursors for the various pituitary hormone-producing cells. This is not without precedent; in pulmonary tissues. Kim, *et al*. [30] observed a reduction in proliferation of the embryonic *Foxm1* null lung mesenchyme, however proliferation was normal in *Foxm1* null epithelial cells.

During anterior pituitary development, corticotropes arise first, followed by thyrotropes. Somatotropes, gonadotropes and lactotropes terminally differentiate later [43]. We did not assess lactotrope differentiation because prolactin is present in such low levels in the embryo it is very difficult to measure reliably [41]. The fact that corticotropes and thyrotropes exhibit an upward trend, although statistically insignificant, while somatotropes and gonadotropes are reduced in the absence of *Foxm1* null mice suggests a temporal requirement for FOXM1 during pituitary development, and a possible shift in the pituitary cell population toward the cells that differentiate early in pituitary ontogeny, namely the corticotropes and thyrotropes and away from cells that differentiate later, somatotropes and gonadotropes. It is unlikely that these small changes in hormone expression would affect embryonic development or growth and fertility of these mice had they been able to live to adulthood.

Lineage commitment and differentiation of cells occur in a sequential manner and are orchestrated by combinatorial expression of cell type-specific transcription factors and epigenetic modifications. Thus, it is possible that FOXM1 contributes to differentiation of hormone-secreting cell types in the developing pituitary gland rather than or in addition to their proliferation. Towards this end, recent work has highlighted that FOXM1 facilitates the differentiation of bronchiolar epithelial cells in mice and primary neuronal differentiation in *Xenopus* [44]. The reduction in somatotropes and gonadotropes that we observe in *Foxm1* ^*-/-*^ embryos could be due to misregulation of transcription networks that are involved in the differentiation of these cell types in the absence of FOXM1. It is known that *Foxm1* is required for β-catenin activation and the expression of Wnt target genes [45]. β-catenin has recently been shown to play a critical role in a multicomponent transcriptional network required for regulated expression of *Cga* and *Lhb* in the gonadotrope [46]. In somatotropes, β-catenin is important in maintaining homotypic cell to cell contacts. Reduction of β-catenin correlates with disruption in the organization of the somatotrope cell network [47]. The inability of somatotropes to effectively communicate with one another and initiate coordinated and appropriate signal transduction could contribute to reduced GH expression seen in our model.

We conclude that FOXM1 is required for normal somatotrope and gonadotrope numbers and for normal pituitary size. The reduction in cell numbers and pituitary size may be due to effects on proliferation of pituitary precursor cells that are too subtle to be detected using our current methodology. Alternatively, the reduction in the number of somatotrope and gonadotrope cells could indicate a role for FOXM1 in differentiation of these cell types. The effects of FOXM1 on pituitary cell number and/or differentiation may be partially compensated for by other factors and is a focus of our ongoing work. Identification of these factors in the future will help elucidate the comprehensive role of FOXM1 in pituitary development.
